# Salmonellosis as a One Health–One Biofilm Challenge: Biofilm Formation by *Salmonella* and Alternative Eradication Strategies in the Post-Antibiotic Era

**DOI:** 10.3390/ph19010061

**Published:** 2025-12-27

**Authors:** Michał Małaszczuk, Aleksandra Pawlak, Paweł Krzyżek

**Affiliations:** 1 Department of Microbiology, Faculty of Medicine, Wroclaw Medical University, 50-368 Wroclaw, Poland; pawel.krzyzek@umw.edu.pl; 2 Department of Microbiology, Faculty of Biological Sciences, University of Wroclaw, 51-148 Wroclaw, Poland; aleksandra.pawlak@uwr.edu.pl

**Keywords:** *Salmonella*, reptile-associated salmonellosis, zoonoses, biofilm, biofilm eradication, multidrug resistance, alternative therapies

## Abstract

Non-typhoidal *Salmonella* (NTS) are globally distributed zoonotic pathogens of major concern within the One Health–One Biofilm framework. Fluoroquinolone-resistant *Salmonella* strains are included by the World Health Organization (WHO) in the Bacterial Priority Pathogens List as high-risk agents. A key virulence determinant of *Salmonella* is its ability to form biofilms, which may display multidrug-resistant (MDR) characteristics and contribute to bacterial persistence and treatment failure. Animals, particularly poultry and reptiles, represent important reservoirs of *Salmonella*, and reptile-associated salmonellosis (RAS) may manifest as extraintestinal infections in humans. In the post-antibiotic era, there is an urgent need to identify effective alternatives to conventional therapies. This review summarizes current knowledge on *Salmonella* biofilms, with particular attention to their MDR potential, and discusses possible strategies for their prevention and eradication, including specific immunoprophylaxis, bacteriophage therapy, and alternative antimicrobials. The promising antimicrobials include plant-based compounds/extracts, bacteriocins, fatty acids, and synthetic/semi-synthetic substances. The integration of vaccination, phage therapy, and novel anti-biofilm compounds may provide a sustainable alternative to antibiotics in controlling *Salmonella* infections and aligns with the principles of the One Health approach.

## 1. Introduction

Zoonoses constitute infectious diseases developing in humans as a result of exposure to microorganisms that constitute the natural microbiome of animals. Transmission can occur through both direct and indirect contact with these pathogens [1]. Despite the availability of preventive measures, zoonotic infections remain a major public health concern. The recent COVID-19 pandemic clearly demonstrated the serious and multidimensional implications associated with the emergence of zoonotic agents in human populations [2]. The risks associated with the spread of pathogens with zoonotic potential are recognized by the World Health Organization (WHO). This institution reports that approximately 60% of all human infectious diseases are zoonotic in origin, while about 75% of new or emerging infectious diseases arise from animals [3]. Bacterial zoonotic agents pose particular therapeutic challenges due to their multiple virulence factors, the ability to form biofilms, potential for developing antibiotic resistance, and their capacity to efficiently colonize new hosts. Among zoonotic bacteria, *Salmonella,* as a member of the order Enterobacterales, is of particular concern. In the Bacterial Priority Pathogen List (BBPL 2024), the WHO identified *Salmonella* among the most significant threats [4]. The two highest-priority categories, critical and high risk, include Enterobacterales resistant to third-generation cephalosporins or carbapenems, as well as typhoidal and non-typhoidal serovars of fluoroquinolone-resistant *Salmonella*. The growing prevalence of resistant strains highlights the urgent need to develop and implement alternative antimicrobial approaches. According to a report of the Centers for Disease Control and Prevention, during the peak of the COVID-19 pandemic (2021–2022), the number of infections caused by antibiotic-resistant bacteria increased by approximately 20% [5].

**Figure 1:**
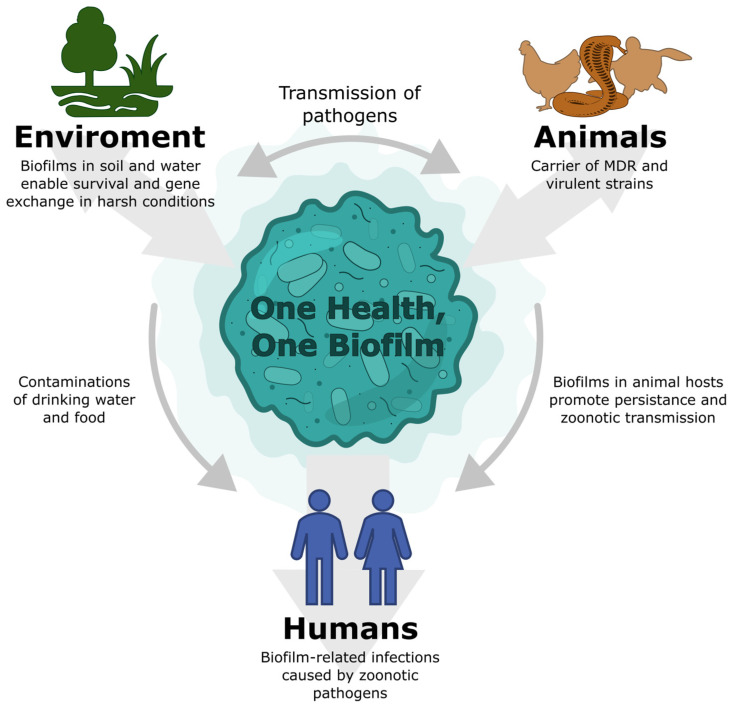
Concept of the One Health–One Biofilm approach.

*Salmonella* pose a significant challenge and remain one of the major focuses within the One Health framework. The One Health initiative is multifaceted and assumes that human health and well-being are closely connected with the health and condition of animals. It also recognizes the importance of the environment in which we live and all the factors that shape it. It addresses not only infectious threats but also non-infectious hazards such as environmental pollution, pesticide exposure, deforestation, and the degradation of diverse ecosystems. Within these environments, One Health highlights potential microbiological risks arising from human interference with wildlife habitats, as well as the contamination of food, recreational areas, and water systems. The concept also promotes a rational and evidence-based approach to animal husbandry, including prudent antimicrobial use, which is considered one of its key components [6,7]. This initiative draws particular attention to the increasing antimicrobial resistance (AMR) of bacterial strains and factors such as the overuse of antibiotics in agriculture and animal husbandry, human-driven expansion into wildlife habitats, climate change leading to animal migration, and the bacterial ability to form biofilms [8,9,10]. The One Health–One Biofilm concept places the biofilm at the core of this relationship, positioning it as a biological link connecting humans, animals, and the environment (Figure 1). The pathogenic potential of *Salmonella* is closely associated with numerous virulence determinants possessed by these bacteria. Among these traits, biofilm formation represents a key virulence factor that significantly enhances bacterial survival both within the host and in the environment. Importantly, it provides protection against antimicrobial factors, including antibiotics. The biofilm also serves as a reservoir of planktonic cells capable of expressing virulence factors that facilitate infection of subsequent hosts.

## 2. *Salmonella* as a Zoonotic Pathogen

*Salmonella* is a rod-shaped, Gram-negative bacterium belonging to the family *Enterobacteriaceae* within the order Enterobacterales. The *Salmonella* genus comprises two species, *Salmonella enterica* and *Salmonella bongori*, although only the former is of significant pathogenic importance to humans. *Salmonella* exhibits substantial variability in its surface structures, leading to the identification of more than 2600 distinct serological variants (serovars). The majority of these serovars belong to *S. enterica* subsp. *enterica* [11]. The antigenic patterns of individual *Salmonella* serovars are determined based on variations in the somatic O antigen (a component of lipopolysaccharide (LPS)), the flagellar H antigen (which may occur in one or two phases), and the Vi capsular antigen [12]. Among all *Salmonella* serovars, two main groups are distinguished: typhoidal *Salmonella*, which includes *S*. Typhi and *S.* Paratyphi, and non-typhoidal *Salmonella* (NTS), which includes all serovars other than these two (mostly *S.* Typhimurium and *S.* Enteritidis). As the NTS serovars constitute a part of the animals’ intestinal microbiota, only these bacteria are etiological factors of zoonotic infections. Animals are the main reservoir of NTS, where these bacteria form a natural part of the intestinal microbiota. Colonization occurs in both wild and kept animals. The highest carriage rates are observed in poultry and reptiles, where up to 80% of individuals within a population may carry *Salmonella* [13,14,15,16], while the occasional colonization of mammals or amphibians have also been detected [17].

### 2.1. Salmonellosis

Salmonellosis is a foodborne disease primarily affecting the gastrointestinal tract. In the United States alone, the annual economic burden of foodborne illnesses has been recently estimated at approximately USD 75 billion, with salmonellosis accounting for USD 17.1 billion. This makes it one of the priciest foodborne pathogen worldwide [18]. Most salmonellosis cases are self-limiting and do not require antibiotic treatment or hospitalization [19]. Due to the often mild symptoms and its self-resolving nature, the actual number of cases is highly underestimated. In some cases, however, salmonellosis can progress to an invasive form. Invasive salmonellosis is caused by the same *Salmonella* serovars responsible for gastrointestinal infections, but primarily affects high-risk individuals, leading to conditions such as meningitis, arthritis, aneurysms, wound infections, or sepsis. In cases of invasive disease, antibiotic treatment and hospitalization are required, as untreated infections may result in death. The most common carriers of *Salmonella* are poultry (mostly broiler and laying and breeding hens). Human infections most frequently occur through consumption of contaminated poultry-derived foods, such as meat, eggs, ice cream, pastries, and ready-to-eat salads. The EFSA and ECDC (European Food Safety Authority and European Centre for Disease Prevention and Control) routinely identify the sources and serovars of salmonellosis in their annual reports. These reports consistently indicate that the poultry production sector represents one of the major sources of this foodborne pathogen in Europe [20,21]. Both the One Health perspective and EFSA reports consistently identify the poultry sector as a critical component in the epidemiological chain of salmonellosis. *Salmonella* is present at numerous key points of the poultry production continuum, and contamination can occur at multiple stages. Potential sources include contaminated feed and water consumed by chickens, as well as the persistence of the pathogen on inanimate surfaces such as incubators, drinkers, and other equipment. The high prevalence of *Salmonella* in poultry flocks is further driven by transmission through vector organisms, by spillover from wild birds, and by vertical transmission within breeding lines [22,23]. Each year, numerous *Salmonella* outbreaks are confirmed worldwide, emphasizing the continuing public health relevance of foodborne transmission [24,25,26].

In addition to the foodborne route, infection may also occur through direct or indirect contact with animals. This is partly related to the ability of *Salmonella* to form biofilms, which enhances bacterial survival in the environment outside the host organism. *Salmonella* contamination has been detected in the immediate surroundings of animals, such as on stair railings, sinks, and household vacuum cleaners in homes with animal reservoirs, as well as on zoo infrastructure [27,28]. Reptiles represent the primary animal source of *Salmonella* transmission to humans through non-foodborne routes, while infections linked to other animals, such as kept dogs, cats, or rodents, are sporadic and rarely documented. This highlights the unique epidemiological significance of reptiles and biofilm forms of *Salmonella* in its zoonotic, extraintestinal transmission.

### 2.2. Reptile-Associated Salmonellosis

Reptile-associated salmonellosis (RAS) represents a distinct form of infection caused by NTS originating specifically from reptiles. These infections often exhibit an extraintestinal course and occur in both high-risk patients and otherwise healthy individuals. If left untreated, RAS may progress to multiorgan failure and ultimately death. Although epidemiological data on RAS are not systematically collected in many countries, numerous reports confirm the reptilian origin of *Salmonella* isolates (Table S1) [29,30,31,32,33,34,35,36,37]. The causes of reptile colonization by *Salmonella*, particularly by serovars characteristic of the subspecies *arizonae* and *diarizonae*, have not been clearly explained. Among the possible factors, a high capacity for biofilm formation of these variants is suggested. Biofilm formation enables *Salmonella* to persist for extended periods in the environment outside the host, which explains why RAS infections often occur through indirect contact with reptiles, e.g., via contaminated water or objects [36,37,38]. Although these strains are rarely isolated, they remain capable of expressing virulence factors under challenging environmental conditions, exhibiting cell invasiveness, resistance to selected antibiotics (including multidrug resistant (MDR) phenotypes), and resistance to the bactericidal activity of the complement system in vitro [39,40,41,42,43].

### 2.3. Drug Resistance in Salmonella enterica

*Salmonella* exhibits a broad spectrum of antibiotic resistance. As mentioned above, fluoroquinolone-resistant *Salmonella* strains are considered priority pathogens by the WHO. Resistance to fluoroquinolones is primarily mediated by the presence of plasmid-encoded *qnr* genes, or *oqxAB* and *qepA*, both of which encode quinolone efflux systems. Alternatively, it can be mediated by point mutations in *gyrA*, *gyrB*, and *parE,* altering the quinolone targets: DNA gyrase and topoisomerase IV [44].

Beyond fluoroquinolones, antibiotic resistance of *Salmonella* results from the presence of various classical resistance genes. These include aminoglycoside acetyltransferase genes conferring resistance to aminoglycosides; *tetA*, *tetB*, and *tetC* responsible for tetracycline and tigecycline resistance; and *bla_LAT-1_*, *bla_CTX_*, *bla_OXA_*, or *bla_TEM_* family genes associated with resistance toward β-lactams, as well as *sul1–3* leading to sulfonamide resistance [44]. In addition, the AcrAB–TolC efflux system contributes to decreased susceptibility to many antibiotics, including tigecycline and fluoroquinolones [45].

An even more important mechanism than monoresistance is MDR—simultaneous non-sensitivity to at least one agent in three or more classes of antibiotics [46]. The most commonly isolated NTS serovars, *S.* Enteritidis and *S.* Typhimurium, often exhibit MDR phenotype [47,48]. Such strains frequently reside in ready-to-eat meat products and poultry farm environments. Alarmingly, a high prevalence of MDR *Salmonella* has been reported in Asia—including 81.8% MDR strains isolated from retail chicken outlets and 91% from broiler samples [49,50]. However, the problem is not limited to Asia region. Ćwiek et al. confirmed the presence of MDR *S.* Enteritidis in both poultry and human fecal samples in Poland. The most resistant isolate displayed resistance to five antibiotics (nalidixic acid, chloramphenicol, colistin, tetracycline, and co-trimoxazole). Notably, colistin resistance was confirmed in all identified MDR profiles [51]. Even more alarming results were presented by Parvin et al., who identified that 17.6% of extended-spectrum beta-lactamase-positive (ESBL+) *Salmonella* strains exhibited MDR to 9–11 antibiotic classes [52]. MDR *Salmonella* strains are also isolated from non-poultry sources, including pork retail outlets, slaughterhouses, pigs, and goat meat [53,54,55]. Importantly, the MDR phenotype frequently coincides with biofilm production, which further complicates bacterial eradication and control [56,57,58].

### 2.4. Salmonella Virulence Factors

The pathogenic potential of NTS results from the presence of numerous virulence factors encoded by this bacterium. The virulence genes are primarily located within *Salmonella* Pathogenicity Islands (SPIs), which can be acquired and transferred through horizontal gene transfer during evolution. The most important of these islands are SPI-1 and SPI-2, whose gene products are essential for intestinal colonization and disease development [59,60,61]. SPI-1 genes are primarily involved in the formation of the Type III Secretion System (T3SS), which functions as a “molecular needle” used to deliver effector proteins directly into host cells. This system determines the efficiency of epithelial cell colonization and promotes bacterial internalization and proliferation [62,63]. Within SPI-2, the genes encode effector proteins responsible for intracellular survival, cytoskeletal rearrangement and formation of the *Salmonella*-containing vacuole, which enables bacterial persistence within phagocytes [64]. Interestingly, apart from the direct cytotoxicity against host cells, genes involved in biofilm formation may also be located within pathogenicity islands [65]. Moreover, the high activity of the SPI-1-based T3SS also plays an important role in the biofilm formation [66].

## 3. Biofilm Formation by *Salmonella*

### 3.1. Regulation of the Biofilm Development

As emphasized several times before, one of the key features of *Salmonella* that enables colonization of the host is its ability to produce biofilm. Biofilms are multicellular microbial structures, often referred to as “microbial communities”. These structures can be formed by single or multiple species of bacteria or fungi and are organized into multilayered, heterogeneous assemblies embedded within an extracellular matrix (ECM). Moreover, the presence of ECM and the close spatial arrangement of microbial cells within the biofilm enhance genetic mutation rates and increase tolerance to environmental stressors (e.g., by upregulation of efflux pumps), thereby contributing to the development of antibiotic resistance. *Salmonella* forms biofilms on various biotic and abiotic surfaces like glass, plastic, stainless steel plants or gallstones [67,68,69]. Multicellular-like behaviors in microorganisms, such as metabolic differentiation and biofilm formation, are tightly coordinated through quorum sensing (QS) mechanisms that allow cells to assess population density. In *Salmonella*, the primary QS signal is the LuxS-dependent autoinducer-2 (AI-2) [69,70,71]. The QS is associated with several key microbial functions: adhesion, development of the biofilm, response toward antibiotics, immune responses, and other antimicrobial agents, as well as facilitation of horizontal gene transfer [72]. In addition to AHL molecules, QS signals may also include diffusible signaling factors (DSFs), fatty acid-based compounds, whose diversity, modes of biosynthesis, and regulatory significance are still being uncovered [73]. Beyond AI-2 and DSF–mediated communication, biofilm development in *Salmonella* is strongly regulated by the intracellular second messenger cyclic di-GMP (c-di-GMP). Fluctuations in c-di-GMP levels modulate the activity of the biofilm regulator CsgD and induce the expression of genes encoding extracellular matrix components. The c-di-GMP regulatory system is not unique to *Salmonella*, but occurs broadly across bacteria such as *Pseudomonas* [74]. This regulatory network is further integrated with global transcriptional control mediated by the alternative sigma factor RpoS, which coordinates the transition to stress-resistant and biofilm-associated states [75,76,77,78].

### 3.2. Biofilm Metrix Composition

Depending on the microbial species involved, the ECM of *Salmonella* is composed of extracellular or cell-associated components, such as polysaccharides (e.g., alginate, α-mannans, β-glucans), proteins (fibronectin-binding proteins, biofilm-associated proteins [BAP], lectins, pilins), nucleic acids (extracellular DNA and RNA), and lipid components (including glycerolipids, teichoic and lipoteichoic acids, and LPS) [72]. Although the *Salmonella* biofilm is multi-component, its ECM is composed primarily of proteins and polysaccharides; however, the quantitative and qualitative proportions of these components vary depending on the strains analyzed and their cultivation conditions.

The major ECM proteins in *Salmonella* biofilms are curli fimbriae and BAP. Curli fimbriae are highly conserved structural elements also found in other *Enterobacteriaceae*, particularly in *Escherichia coli* [73]. Curli fimbriae are pathogen- and biofilm-associated proteins involved in surface adhesion and formation of multicellular aggregates, which can facilitate cross contamination of food and host cell invasion [75,76,77]. Structurally, curli fimbriae are amyloid fibers assembled through the activity of a type VIII secretion system (T8SS) [77]. The genes encoding curli fimbriae are organized in the *csgBAC* and *csgDEFG* operons. The structural subunits are encoded by *csgA* (major subunit) and *csgB* (minor subunit), while the remaining genes act as periplasmic chaperones, transcriptional activators, and form the secretion–assembly machinery [77]. The second major protein component of the *Salmonella* biofilm ECM is BapA (biofilm-associated protein A) [79]. BapA is a large, conserved secreted protein shown to be essential for the formation of mature biofilm structures in *Salmonella* and contributing significantly to the efficiency of the host colonization [78,79].

A crucial polysaccharide component of the *Salmonella* biofilm matrix is cellulose, a long polymer of β-(1→4)-linked D-glucose units. The cellulose biosynthesis operon (*bcs*) in *Salmonella* includes *bcsA*, *bcsB*, and *bcsC*, which are directly involved in polymer synthesis. Additionally, *bcsEFG*, *bcsQ*, *bcsR*, and *bcsZ* serve regulatory roles, mediating the processes of cellulose synthesis and extracellular secretion. Cellulose synthesis and biofilm formation are regulated by the intracellular level of cyclic dimeric (3′→5′) GMP (c-di-GMP) in bacterial cells [80]. It has been shown that c-di-GMP level, cellulose synthesis, and consequently the formation of cellulose-containing biofilm, may lead to reduced virulence of the strains while promoting transmission and asymptomatic colonization of the host [81]. This effect is linked to inhibition of virulence gene expression mediated by *bcsA* [82].

The production of the main *Salmonella* biofilm components—cellulose and curli fimbriae—can be indirectly observed using the Congo Red Agar (CRA) method. The appearance of individual colony types on CRA depends on the type and intensity of ECM component production. Two general morphotypes can be distinguished: rough and smooth. Rough biofilm morphotypes are characterized by increased synthesis of one or both ECM components. *Salmonella* typically displays three rough morphotypes: RDAR (red, dry, and rough), PDAR (pink, dry, and rough), and BDAR (brown, dry, and rough), which are result of an increased ECM production. RDAR colonies produce both cellulose and curli fimbriae, PDAR colonies produce cellulose only, while BDAR colonies produce curli only [83,84]. When ECM production decreases, *Salmonella* exhibits smooth colony morphotypes—ras (red and smooth), pas (pink and smooth), and bas (brown and smooth)—which correspond to the same ECM components as their rough variants. An additional saw colony morphotype (smooth and white) is observed in strains lacking ECM production, indicating the absence of biofilm formation in the tested conditions.

**Figure 2:**
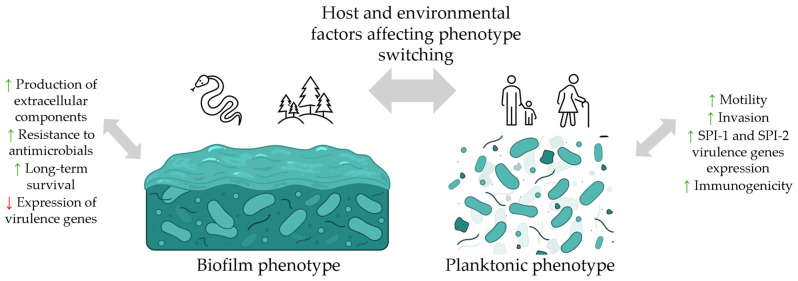
Biofilm–planktonic phenotype switching in *Salmonella* and the resulting phenotypic outcomes. Legend: ↑, increase; ↓, decrease.

Importantly, studies have shown that the process of biofilm formation and the development of distinct CRA morphotypes are temperature-dependent. A single isolate may exhibit different CRA morphotypes depending on the incubation temperature. Enhanced biofilm production and the appearance of rough morphotypes are observed at lower temperatures (3 °C to 28 °C), which promotes the persistence of virulent and antibiotic-resistant strains in the external environment [84,85,86]. The temperature-dependent biofilm formation and the variability of morphotypes observed on CRA indicate a phenotypic switch in *Salmonella* from the biofilm-associated with the planktonic state. Increased biofilm formation at lower temperatures is accompanied by reduced flagellar motility observed at 28 °C compared to 37 °C, as demonstrated for, e.g., *S.* Typhimurium, *S.* Erlangen and *S.* Isaszeg [87]. This phenomenon emphasizes the ecological role of biofilm formation as a mechanism facilitating the persistence and transmission of virulent *Salmonella* beyond the host, serving as a link between animal reservoirs and human populations (Figure 2).

### 3.3. Experimental Techniques to Study Biofilm of Salmonella

Biofilm science has been an active research area since the late 1970s, when biofilms were first formally defined. Since then, advances in technology have significantly reshaped our understanding of biofilms. In vitro biofilm models can be highly customized to replicate specific systems. The simplest approach is the microtiter plate assay, in which biofilms grow on the microplate surface and are either stained with crystal violet to measure total biomass or transferred onto agar plates to assess cell viability. More recently, quantification of viable biofilm cells using qPCR has been proposed as an alternative. To support biofilm maturation and address nutrient limitations, more sophisticated setups such as continuous-flow chambers and microfluidic devices are currently also used. As with model systems, imaging techniques range from basic, low-resolution methods to advanced, high-resolution microscopy. Light microscopy provides a simple means of confirming the presence of biofilms, while fluorescence microscopy—using dyes such as SYTO-9, propidium iodide, or ECM-specific fluorophores—enables assessment of cell viability and visualization of ECM components. Fluorescence imaging often precedes more detailed structural analyses conducted with advanced techniques like scanning electron microscopy (SEM) and transmission electron microscopy (TEM), which allow for ultrastructural imaging of microorganisms within a biofilm and their interaction with the substrate surface [88,89,90].

On the technical side, research on *Salmonella* biofilms closely mirrors that conducted on other microorganisms. Most studies rely on standard biofilm assessment techniques, such as crystal violet staining to quantify biofilm biomass [91,92,93,94,95,96] or plating methods to determine the viability of biofilm-associated cells [93,97,98,99]. As outlined in the previous section, analyses of the chemical composition of *Salmonella* biofilms are often complemented by evaluating colony morphotypes using a Congo Red staining [94,95,100]. Some researchers further investigate biofilm structure through confocal laser scanning microscopy (CLSM) [95,101,102] or SEM [94,95,99,101]. Moreover, in the last few years, the use of Fourier transform infrared spectroscopy (FT-IR) and Raman spectroscopy methods in the analysis of *Salmonella* biofilm biochemistry has been gaining importance [96,103]. While biofilm formation of *Salmonella* is typically examined in conventional plastic microtiter plates, certain research groups expand their work to food ex vivo models, assessing biofilm development on food (meat or eggs) or food-contact surfaces (stainless steel, silicone, and nylon) [57,104,105]. Currently, *Salmonella* biofilm studies are performed almost exclusively under static (non-flow) conditions, while the use of flow-based systems—common in studies of other microorganisms—remains rare in this field [98,106,107].

## 4. Methods for the *Salmonella* Biofilm Prevention and Eradication

**Figure 3:**
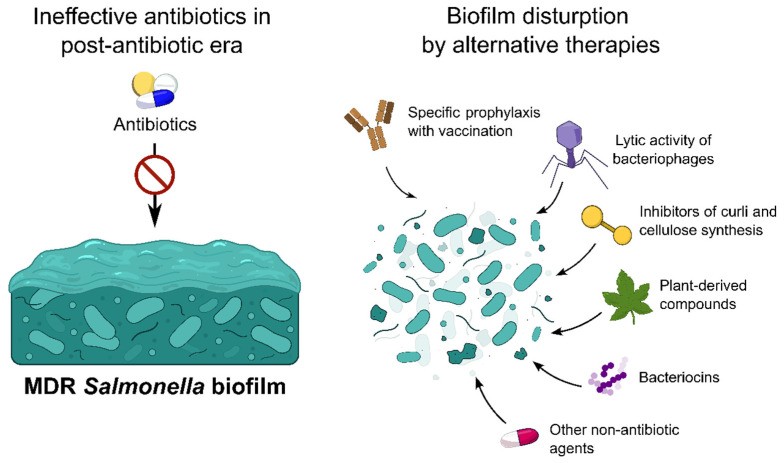
Alternative strategies applicable to multidrug-resistant biofilms of *Salmonella*.

Considering the increasing antimicrobial resistance of bacterial strains, it is crucial to develop alternative approaches for the eradication of difficult-to-treat infections. Biofilm formation greatly enhances the resistance of bacterial populations to antibiotics, and MDR *Salmonella* biofilms have already been reported in the literature. Gao et al. observed a high level of biofilm production in MDR *S.* Typhimurium [108]. According to Locke et al., high percent of the *Salmonella* isolated from clinically diseased animals have ability for strong biofilm production [109]. Bhardwaj et al. reported that over 80% of strong to moderate biofilm-producing *S.* Typhimurium isolated from poultry meat were MDR [110]. For this reason, alternative strategies for biofilm eradication as a method of limiting the MDR phenomenon are being intensively explored by scientists (Figure 3).

### 4.1. Vaccination

The most effective method of preventing infectious diseases is vaccination. A vaccine is a biological preparation designed to safely trigger an immune response that protects against infection or disease. In most vaccines, the key elements are one or more antigens that stimulate protective immune responses [111]. It is not only effective but also economically beneficial, as preventing disease is far more cost-effective than treating it. Thanks to vaccinations, the worldwide number of cases of different live-threatening diseases has been drastically reduced or has even been completely erased. Vaccinations, by preventing the development of infectious diseases, also constitute a promising mode to fight biofilm-mediated tolerance and/or resistance to antibiotics [112,113]. To date, unfortunately, no vaccine effective against invasive NTS (iNTS) is commercially available. While a vaccine against typhoid fever exists, iNTS continues to claim a deadly toll in many countries (particularly in sub-Saharan Africa [SSA]). Risk groups with increased mortality include children under 5 years of age, adults over 70 years of age, and immunocompromised patients. Noteworthy, iNTS strains caused 535,000 cases worldwide in 2017, of which 77,500 were fatal. Within all these cases, 421,600 of them occurred in SSA [114]. In line with this, in areas where sociodemographic development is insufficient vaccination against iNTS could save thousands of lives.

Currently, the greatest hopes are associated with the research conducted by Chen et al., who presented the results of a phase 1 clinical trial of the Trivalent *Salmonella* conjugate vaccine (TSCV) [115]. The authors indicated that nearly 90% of iNTS cases in children under 5 years of age are caused by serovars *S.* Typhimurium (and other serovars belonging to Group O:4 (B)), and *S*. Enteritidis (and other serovars belonging to Group O:9 (D)). Therefore, the TSCV formulation includes the core and O-specific polysaccharides (COPS) from *S.* Typhimurium and *S.* Enteritidis, linked to Phase 1 flagellins. This is additionally combined with the *S.* Typhi Vi conjugate (Typbar TCV™), the first WHO-prequalified conjugate supported by GAVI. In phase 1 of clinical trials, this vaccine showed high efficacy and was well tolerated by the volunteers participating in the study, so subsequent phases of clinical trials were planned.

Another promising discovery is the bivalent iNTS-GMMA vaccine, which has undergone its first human dose escalation trials [116]. The vaccine against two *Salmonella* serovars: *S.* Typhimurium and *S*. Enteritidis, is based on Generalized Module of Membrane Antigen (GMMA) technology. It utilizes outer membrane vesicles that present bacterial surface proteins and LPS. Studies have demonstrated preliminary safety and efficacy, leading to the qualification of iNTS-GMMA for phase 1 human clinical trials.

It should be noted that the above studies only cover two serovars, the most common causes of iNTS. Even if these vaccines are successfully registered and introduced to the market, they will not provide protection against non-invasive NTS caused by most *Salmonella* serovars. Consequently, transmission of these serovars would remain unaffected, and the vaccines’ impact on biofilm eradication will be limited. Furthermore, the primary role of vaccinations is to prevent severe infections and disease complications, but their effectiveness in combating colonization is only partially effective. The cost of vaccinations should also be considered, as they are a significant social problem in SSA countries.

Both of the vaccines described above are designed to prevent iNTS and, therefore, may arrest potentially the development of biofilms of these bacteria. Unfortunately, they do not have the potential to eradicate preformed *Salmonella* biofilms. To achieve this, a vaccine containing biofilm-specific antigens rather than antigens from planktonic bacterial forms would need to be developed. This goal is difficult to achieve due to the specific structure of biofilms and the high variability of these structures [117]. Thanks to the development of vaccinology techniques combined with the use of artificial intelligence, studies on vaccines directly targeting biofilms of various bacterial species are conducted [78,116]. Human vaccines against bacterial biofilms pose a challenge for modern vaccinology and will be intensively studied in the coming years [118].

### 4.2. Bacteriophages

Bacteriophages are viruses capable of infecting bacterial cells, leading to genetic alterations within bacterial cells and, in most cases, cell lysis. They are present in nearly all ecological niches, with an estimated abundance of ~10^31^ virions globally [119]. This vast abundance, combined with the remarkable evolutionary adaptability of bacteriophages, highlights their significant potential as biological agents to counteract the increasing prevalence of antibiotic resistance. In recent years, phages have been extensively investigated as alternative or complementary antimicrobial strategies, both for the treatment of infections caused by antibiotic-resistant bacteria and for the eradication of biofilm-associated cells in environmental and clinical settings. The antimicrobial efficacy of bacteriophages is primarily attributed to their ability to encode a diverse repertoire of enzymatically active proteins, including lytic enzymes, endolysins, holins, depolymerases, peptidases, or muramidases, targeting capsular and biofilm polysaccharides and cell wall components [50,120,121]. They have been applied for the treatment and eradication of a broad range of Gram-positive and Gram-negative bacteria, including members of Enterobacterales. Moreover, the efficacy of bacteriophages in disrupting and eliminating *Salmonella* biofilms has been consistently demonstrated in numerous in vitro studies over the past few years [122,123,124,125,126,127,128,129,130,131,132,133]. It has been shown that this process depends on environmental conditions; therefore, proper characterization of potential phage preparations is crucial [122].

Jaglan et al. [123] tested the lytic activity of phage phiSalP219 against multiple *Salmonella* strains. They demonstrated high lytic efficacy against MDR *S.* Paratyphi, as well as moderate activity against MDR *S*. Enteritidis and *S*. Typhimurium (Efficiency of Plating (EOP) values of 1.0, 0.25, and 0.1, respectively). The researchers also confirmed the phages’ effectiveness in eradicating matured formed by MDR *S.* Typhimurium and *S.* Paratyphi. The lytic activity against biofilms formed by MDR *Salmonella* Thompson and *Salmonella* Mbandaka was demonstrated by Park et al. [124]. A significant reduction in *Salmonella* biofilm biomass and the efficacy of the *Epseptimavirus* phage MSP1 in food preservation applications were confirmed in this study. Moreover, the anti-biofilm potential of other *Epseptimavirus* phages against *Salmonella* has also been reported in recent years [125]. The lytic activity of bacteriophages is influenced by environmental temperature, underscoring the importance of a comprehensive phage characterization and the selection of optimal application conditions for their effective use in biofilm eradication [84].

Phages have also been tested against *Salmonella* isolated from reptiles. One such strain *S.* Blockley Sal-SNUABM-svn1, obtained from a cloacal swab of a Savannah monitor (*Varanus exanthematicus*), was used for the isolation of bacteriophage pSal-SNUABM-02 [126]. This phage exhibited EOP values ranging from 0.35 to 1.04 against several reptile-derived *Salmonella* strains, while no lytic activity was detected against poultry isolates, indicating host specificity toward RAS. Moreover, pSal-SNUABM-02 effectively inhibited biofilm formation in vitro and disrupted preformed biofilms on reptile skin surfaces, demonstrating strong potential as a biocontrol agent targeting RAS. The lytic activity against *S.* Enteritidis, *S.* Typhimurium, and *S.* Abortusequi, as well as the depolymerase-mediated degradation of *S.* Abortusequi biofilm, has been demonstrated [127].

It is essential that promising results obtained in laboratory conditions demonstrate equally strong in vivo efficacy and can be successfully adapted within the animal production and food processing chain. In line with this, Korzeniowski et al. demonstrated the in vitro effectiveness of selected bacteriophages, and subsequently confirmed their in vivo activity in controlling *S. Enteritidis* on poultry drinkers [128]. To enhance the effectiveness of preparations and reduce the dose required for cell lysis or biofilm eradication, bacteriophages can also be used in combination with other substances or as phage cocktails containing several viruses. Yuksel et al. demonstrated that phage P22 inhibited biofilm formation by *S.* Typhimurium by 80%. The researchers observed a synergistic inhibitory effect on the biofilm production when the bacteriophage was combined with EDTA and nisin at low concentration levels (phage titer 10^2^ PFU/mL, EDTA 1.25 mM and nisin 9.375 µg/mL) [129]. A promising data was presented by Cong et al. [125], who tested phage cocktails composed of three to six phages for the eradication of mature biofilms and for surface coating against *S.* Typhimurium, *S.* Infantis, *S.* Heidelberg, and other serovars. They noticed that a cocktail of six phages (JC1, S5p2, 29, 52, 1PB, and VCA1) achieved nearly 100% efficacy in eradicating *S.* Typhimurium biofilm formed on stainless steel surfaces. The list of selected studies confirming the anti-biofilm activity of bacteriophages is presented in Table 1.

**Table 1:** Use of different bacteriophages in the control of multiple serovars of *Salmonella* populations and its biofilm eradication.

Phage	Activity	Serovar	Experimental Model	Parametrical Changes	EOP *	Reference
lysSEP21	Lytic	*S.* Enteritidis *S.* Typhimurium	In vitro and food ex vivo model	Biomass ↓ (≤60% in 5 h; in vitro) Viability ↓ (6–9 log_10_ in 5 h; in vitro) Viability ↓ (≤3 log_10_ CFU/mL in 9 days; in vivo)	*ns*	[130]
UPWr_S134 phage	Lytic	*S.* Enteritidis	In vitro and animal in vivo model	Biomass ↓ (≤54% in 4 h; in vitro) Viability ↓ (1–2 log_10_ CFU/mL in 9 days; in vivo) Biomass ↓ (≈60% in 24 h; in vitro)	*ns*	[128]
phage X5	Lytic	*S.* Pullorum	In vitro and food ex vivo model	Viability ↓ (1.5 log_10_ CFU/mL in 24 h; in vitro) Viability ↓ (1–2.5 log_10_ CFU/mL in 24 h; ex vivo)	*ns*	[131]
KE04, KE06, KE15, KE17, KE26, KE24, KE23	Lytic	*S.* Typhimurium, *S.* Enteritidis	In vitro	Biomass ↓ (≤70% in 24 h)	≤3	[132]
P22	Lytic	*S.* Typhimurium	In vitro	Biomass ↓ (≤80% in 24 h)	*ns*	[129]
Epseptimavirus MSP1 phage	Lytic	*S.* Thompson	In vitro and food ex vivo model	Biomass ↓ (≤46.4% in 12 h; in vitro) Viability ↓ (≤3.5 log_10_ CFU/mL in 4 h; in vitro) Viability ↓ (≤5 log_10_ CFU/mL in 3–12 h; ex vivo)	0.1–1	[124]
UPF_BP1 and UPF_BP2 phages	Lytic	*S.* Gallinarum	In vitro	Activity against 78% of biofilm forming isolates and against 77% of MDR strains	*ns*	[122]
Jerseyvirus 4FS1 phage	Lytic and ECM depolymerization	*S.* Enteritidis, *S.* Typhimurium, *S.* Abortusequi	In vitro	Biomass ↓ (≤75% in 2 h)	≤1	[127]
Agtrevirus phages	Lytic	*S.* Blockley	In vitro and animal ex vivo model	Biomass ↓ (≤35% in 48 h; in vitro) Viability ↓ (≤3 log_10_ CFU/mL in 48 h; in vitro) Viability ↓ (≤2.5 log_10_ CFU/mL in 48 h; ex vivo)	1	[126]
**Phage cocktails**						
4 phages cocktail	Lytic	*S. enterica* (I)	In vitro	Strain-dependent biomass ↓ (10–100% in 24 h; in vitro)	≤1 for single phages	[134]
3 phages cocktail	Lytic and ECM depolymerization	*S*. Infantis	In vitro	Condition-dependent viability ↓ (mostly 3–4 log_10_ CFU/mL in 4–8 h)	1–1.46	[84]
3 phages cocktail	Lytic	*S.* Heidelberg	In vitro	Strain-dependent viability ↓ (≤90% in 7 days)	*ns*	[135]
5 phages cocktail	Lytic	*S.* Typhimurium	In vitro	Biomass ↓ (≤63.6% in 24 h)	*ns*	[136]

* *ns*—not specified; EOP—Efficiency of Plating (≥0.5 high, 0.49–0.1 moderate, 0.099–0.001 low, <0.001 non-efficient). Legend: ↓, decrease.

### 4.3. Plant-Based Compounds

Medicinal plants have long been used worldwide for a wide range of therapeutic applications, including the management of microbial infections. In 2022, the global market for herbal medicines was estimated at USD 170 billion, and forecasts indicate it could rise to USD 600 billion by 2033. Growing research attention is now directed toward plant-derived compounds and extracts for their ability to suppress pathogenic microorganisms and enhance the host’s defense against infections [137,138]. Literature suggests that these phytochemicals demonstrate anti-biofilm activity by direct antimicrobial action, disruption of ECM synthesis pathways, and interference with microbial communication systems (QS) [139] (Table 2).

Promising results have been obtained in studies on essential oils and their use in the biofilm eradication. Kim et al. [140] demonstrated that the use of clove essential oil and eugenol effectively inhibited the production of curli fimbriae, resulting in the absence of biofilm formation by *E. coli* O157:H7. While the minimum inhibitory concentration (MIC) of these compounds was 0.01%, anti-biofilm activity was observed at concentrations as low as half of the MIC value. Other essential oils, including clove essential oil and star anise essential oil, inhibited biofilm formation by *S.* Thompson at concentrations of 0.62 μL/mL and 25 μL/mL, respectively. El-Demerdash et al. [141], demonstrated the efficacy of nano-garlic emulsion combined with neomycin in inhibiting biofilm formation through the suppression of *csgA, csgB, csgD* genes. The combined application of the nano-garlic emulsion and neomycin enhanced the overall antibacterial effect, leading to a markedly stronger inhibition of bacterial growth than either agent used individually. Diosmin exhibited both inhibitory and eradication activity against *S.* Typhimurium biofilms [142]. At an 0.5 mg/mL it reduced biofilm formation of the ATCC strain and the clinical strain by 52% and 64%, respectively. For biofilm eradication, it decreased biofilms by 1.2 log and 1.9 log, respectively. The activity of ferulic and p-coumaric acids against *S.* Enteritidis biofilm formation and motility was also confirmed by Xu et al. [143].

### 4.4. Microbial and Host Peptides

Another promising strategy for controlling *Salmonella* biofilms involves the use of antimicrobial peptides (AMPs). AMPs constitute bioactive peptides with antimicrobial and immunomodulatory activities. They originate from diverse sources and are present across all forms of life—from bacteria and plants to vertebrates and invertebrates—where they help limit pathogen spread and support in the regeneration of tissues. Advances in systems and synthetic biology have enabled the artificial production of these peptides and have highlighted them as promising candidates for next-generation antibiotic alternatives [144,145]. The net positive charge, amphipathic nature, and small size enable AMPs to attach to and disrupt microbial membranes, including those within established biofilms. In addition, AMPs modulate the inflammatory response by influencing epithelial and immune cells, thereby enhancing the host’s immune activity against biofilms [146] (Table 2).

Several studies have demonstrated their effectiveness against different *Salmonella* serovars, both in planktonic and biofilm-associated forms. Enterocin AS-48, produced by *Enterococcus faecalis*, exhibited a synergistic effect with antibiotics in inhibiting bacterial growth and removing preformed biofilms at concentrations of 25 µg/mL and 50 µg/mL, respectively [147]. Moreover, *Pediococcus*-derived bacteriocins K10 and HW01 effectively reduced *S.* Typhimurium biofilm formation on stainless steel surfaces and in meat matrices, highlighting their potential application in food safety and processing environments. Bovine lactoferrin and its peptides exhibited strong bactericidal activity (>80% reduction) against planktonic *S.* Typhimurium ATCC 14028 and disrupted >50% of preformed biofilms on abiotic surfaces after 4–6 h of exposure in vitro [148].

### 4.5. Fatty Acids

Antimicrobial lipids are single-chain amphiphilic molecules that interact with microbial membranes and ultimately cause cell lysis. Fatty acids, one of the most extensively studied classes of antimicrobial lipids, consist of a saturated or unsaturated hydrocarbon chain with a terminal carboxylic acid group. In biological systems, they typically contain an even number of carbon atoms, ranging from 4 to 28. Fatty acids shorter than 8 carbons are classified as short-chain, those with 8–12 carbons as medium-chain, while those exceeding 12 carbons as long-chain. Their antimicrobial actions primarily involve disrupting bacterial membranes—through destabilization and pore formation—and interfering with essential protective and functional cellular processes [149]. Moreover, because of their structural resemblance to diffusible signal factors (DSFs), many fatty acids may modify DSF-dependent microbial communication pathways and disrupt the biofilm formation ability [150] (Table 2).

Ng et al. demonstrated that several short-chain fatty acids, including caproic, acetic, isobutyric, and valeric acid, exhibit inhibitory activity against both planktonic cells and biofilms of *S.* Typhimurium and *S.* Enteritidis [151]. In the case of *S.* Typhimurium biofilms, propionate and butyrate were also effective, reducing the biofilm biomass by approximately 50% [152]. For S. Enteritidis, Shah et al. reported similar inhibitory effects of caprylic acid and its nano-emulsion on biofilm formation, with the nano-emulsified formulation exhibiting superior anti-biofilm activity [153].

### 4.6. Synthetic and Semi-Synthetic Compounds

Most licensed antibiotics were derivatives of existing chemical classes and met few of the WHO’s four innovation criteria: introduction of a new chemical class, a novel target, a new mode of action, and absence of cross-resistance with current antibiotics. Today’s development pipeline remains similarly dominated by derivatives of known classes, which present lower development risks, but rarely fully overcome established resistance mechanisms. In contrast, novel synthetic and semi-synthetic therapeutics are especially valuable for tackling AMR, because they often feature new mechanisms of action and/or bind at sites distinct from those targeted by clinically used antibiotics. The value of these compounds may also stem from their capacity to diminish microbial virulence, thereby lowering the selective pressure that drives resistance [154,155] (Table 2).

The *E. coli* model has been widely used to investigate compounds that inhibit the production of curli fimbriae, i.e., one of the most important components of *Salmonella* biofilm. Among these, FN075 and BibC6 demonstrated inhibitory activity against uropathogenic *E. coli* (UPEC) [156]. Andersson et al. [157] confirmed the activity of modified 2-pyridone compounds, which inhibited the expression of *csgA* and consequently suppressed curli fimbriae production in vitro, leading to a significant reduction in the biofilm formation. The researchers introduced multiple structural modifications to the parent compound, resulting in derivatives with varying levels of activity. The strongest inhibition of curli fimbriae polymerization was observed for a ring-fused 2-pyridone derivative in which the peptidomimetic backbone was extended by introducing an amine group. Other innovative approaches for combating *Salmonella* biofilms include the use of plasma-treated materials or symmetrical selenoesters. Plasma-treated water, for instance, has been shown to significantly reduce *S.* Typhimurium biofilm formation at a concentration of 25% [158]. Symmetrical selenoesters, on the other hand, exhibit variable antimicrobial activity and their mechanism of action has been attributed to the efflux pump inhibition [159].

**Table 2:** Selected alternative non-antibiotic approaches for the control of *Salmonella* and its biofilm eradication.

Category	Substance	Strain	Effect	Antimicrobial Values	Reference
Plant-based compounds	Cinnamon essential oil	*S.* Thompson	Disrupting biofilm integrity	0.62 μL/mL	[160]
	Cinnamon star anise essential oil			25 μL/mL	
	Clove oil, Eugenol	EHEC model	Inhibition of curli fimbriae production	0.005%	[140]
	Nano-garlic emulsion	*S.* Typhimurium, *S.* Infantis, *S.* Kentucky, *S.* Molade	Inhibition of biofilm formation by downregulating *csg* genes	12.5–25 μg/mL	[141]
	Diosmin	*S.* Typhimurium	Reduction in biofilm formation	0.5–2 mg/mL;	[142]
	Frulic acid	*S.* Enteritidis	Inhibition of motility, biofilm biomass, and EPS production	1.0 mg/mL	[143]
	P-coumaric acid			0.25–0.5 mg/mL	[143]
Antimicrobial peptides	Enterocin AS-48 (*Enterococcus*)	*Salmonella* spp.	Reduction in biofilm and cell grown inhibition	Synergism with antimicrobials; 25–50 mg/L	[147]
	DF01 (*Lactobacillus*)	*S.* Typhimurium	Inhibition of biofilm production	*ns*	[161]
	K10, HW01 (*Pediococcus*)		Reduction in biofilm on stainless steel and in meat	1.0 mg/mL	[162]
	Bovine lactoferrin and lactoferrin-derived peptides		Reduction in biofilm	1 ≥ 10 µM	[148]
Fatty Acids	Caproic acid, Acetic acid, Isobutyric acid, Valeric acid and others	*S.* Typhimurium, *S.* Enteritidis	Eradication of biofilm and planktonic cells	Dependent of compound tested	[151]
	Propionate and Butyrate	*S.* Typhimurium	Eradication of biofilm	2 mg/mL	[152]
	Caprylic acid nano-emulsion	*S.* Enteritidis		≤0.4%	[153]
Synthetic and semi-synthetic compounds	FN075, BibC6	*E. coli* model	Attenuated virulence in a mouse model of urinary tract infection with UPEC model	≥1.0 mM	[156]
	Modified ring-fused 2-pyridone		Inhibition of *csgA* gene and curli production in vitro	50 mM	[157]
	Plasma treated water	*S.* Typhimurium	Reduction in biofilm	25%	[158]

*ns*—not specified.

### 4.7. Limitations of Alternative Methods Fighting Against Salmonella Biofilms

Although the use of alternative therapies in the prevention and eradication of *Salmonella* biofilms is a very attractive therapeutic direction, many challenges must be taken into account before their widespread application in routine clinical practice.

One such factor is the heterogeneity of biofilms [163], including those formed by *Salmonella* [164,165]. This substantial biochemical and structural complexity is reflected in the increasingly common use of the term ‘matrixome’, which encompasses all components of the biofilm matrix—estimated to hundreds or even thousands of different biomacromolecules [72]. As noted earlier in this review, the composition of *Salmonella* ECM varies depending on prevailing environmental conditions [166]. Consequently, identifying a single antimicrobial compound capable of inhibiting the numerous biosynthetic pathways involved in the ECM development in this bacterium may prove extremely challenging, if not impossible [167].

The second issue worth considering is the discrepancy between conditions achieved in laboratory experiments and those encountered by therapeutics within the host. In the human body, antimicrobial agents must reach the infection site—in the case of *Salmonella*, the intestines [168,169]. Along the way, these substances are exposed to numerous unfavorable factors, such as gastric acid, bile, and degradation by the microbiota [170,171]. For bacteriophages, the host immune system’s capacity to recognize and eliminate them is an additional challenge [172,173]. All of these factors can lead to substantially reduced therapeutic concentrations and, consequently, limited in vivo effectiveness, despite highly promising in vitro results [169,170,171].

Another issue is the widespread myth suggesting that microorganisms are unable to develop resistance to alternative therapies [174,175]. According to the authors of this review, this misconception stems from the tendency of researchers evaluating the antimicrobial efficacy of new compounds to emphasize only the benefits of their use and their presumed superiority over conventional antibiotics. However, scientific evidence demonstrates that microorganisms can indeed develop resistance to anti-QS agents [176,177] and plant-derived compounds [178], as well as acquire mutations that reduce susceptibility to bacteriophages [179]. One of the best-described mechanisms of broad, non-selective resistance involves the activity of efflux pumps or membrane vesicles—structures responsible for exporting various compounds out of the cell [180,181]. Although these mechanisms are classically associated with resistance to multiple classes of antibiotics, the literature indicates that efflux pumps and membrane vesicles can also expel phytochemicals and bacteriophages, respectively [182,183]. Notably, both types of structures are well described in *Salmonella* and may play a key role in limiting the effectiveness of alternative antimicrobial strategies [184,185].

In summary, alternative strategies for combating microorganisms and disrupting their biofilm development represent a highly promising avenue for addressing the growing global challenge of antibiotic resistance. Nevertheless, it is important to recognize that, like antibiotics, new therapeutics are also perceived by microorganisms as a threat and may therefore elicit similar defensive responses. Moreover, there is a clear need for more in vivo studies, as well as rigorously designed in vitro experiments that incorporate diverse research models and conditions mimicking the human environment, in order to accurately assess the efficacy of new anti-biofilm agents against *Salmonella*.

## 5. Conclusions

The ability of *Salmonella* to form biofilms represents a major factor underlying its environmental persistence, host colonization, and resistance to antimicrobials. Non-typhoidal *Salmonella* associated with reptiles (RAS) should be recognized as a potential zoonotic source, posing a risk of extraintestinal infections in humans. The coexistence of biofilm formation and MDR significantly limits the efficacy of conventional therapies. The increasing prevalence of MDR *Salmonella* isolates worldwide constitutes a serious threat to both human and animal health, reducing the effectiveness of available treatment options and facilitating pathogen persistence in environmental reservoirs. Addressing this challenge requires innovative, sustainable, and integrated strategies that go beyond traditional antibiotic use.

Recent advances indicate that specific immunoprophylaxis through vaccination and bacteriophage-based therapy offers promising routes to control *Salmonella* infections while reducing antibiotic dependence. Bacteriophages represent one of the most effective anti-biofilm agents, capable of disrupting mature biofilm structures and inhibiting *Salmonella* growth. In addition to their therapeutic potential, phages may also be applied in food preservation systems to reduce bacterial contamination and prevent the formation of biofilms on food-contact surfaces. Furthermore, several non-traditional compounds, including plant-based compounds like essential oils, phenolic acid, plant-derived flavonoids, antimicrobial peptides, fatty acids and other synthetic and semi-synthetic compounds, have demonstrated the potential to interfere with biofilm development and persistence. The combination of these strategies could enhance antimicrobial efficacy and reduce pathogen transmission across animal reservoirs.

Future research should focus on understanding the molecular mechanisms of biofilm formation under environmental and host-associated conditions, particularly in reptile and poultry environments, and on evaluating the in vivo effectiveness of vaccines, bacteriophages, and novel anti-biofilm compounds as part of integrated One Health interventions.

## Data Availability

No new data were created or analyzed in this study.

## Supplementary Materials

The following supporting information can be downloaded at: https://www.mdpi.com/article/10.3390/ph19010061/s1, Table S1. Case reports of reptile-associated salmonellosis.
