# Supplementary material for: Salmonellosis as a One Health–One Biofilm Challenge: Biofilm Formation by Salmonella and Alternative Eradication Strategies in the Post-Antibiotic Era
Source: Pharmaceuticals (Basel). 2025 Dec 27;19(1):61. doi: 10.3390/ph19010061 (PMC12845418; doi:10.3390/ph19010061)
Supplement: Supplementary file 1 [file pharmaceuticals-19-00061-s001.zip › pharmaceuticals-3978172-supplementary.pdf]

Table S1. Case reports of reptile-associated salmonellosis.

Salmonella serovar	Strain's source	Clinical form	Reference
Saintpaul (I)	*Elaphe climacophora*	Testicular necrosis	[29]
Saintpaul (I)	Lizard[1]	Testicular seminoma with testicular abscess	[30]
Oranienburg (I)	*Thamnophis* sp.	Urinary tract infection	[31]
O:38:r:- (IIIb)	Tutrle habitat[1]	Otitis	[36]
Vitkin (I)	*Pogona* sp.	Gastroenteritis	[32]
Kingawba (I)	Reptiles in household[1]	Meningitis	[33]
Salmonella IIIa	Snake in household[1]	Gastroenteritis[2]	[34]
Salmonella IIIa	Not indicated[3]	Sepsis and diarrhea	[35]
Salmonella IIIa	Rattlesnake pills	Sepsis[2]	[36]
Salmonella IIIa	Rattlesnake meat	Gastroenterititis	[36]
Salmonella IIIa	*Pantherophis guttatus*	Osteoarticular	[37]

(1) species not indicated; (2) fatal infections; (3) direct contact with a reptile was not confirmed in this case, however, infection with a *Salmonella* isolate typical of RAS suggests indirect exposure to bacteria persisting in the environment outside the host.
